# Determination of Blasting Vibration Safety Criterion for HDPE Pipeline Using Vibration and Strain Data in a Coastal Metro Line

**DOI:** 10.3390/s21217252

**Published:** 2021-10-31

**Authors:** Nan Jiang, Yuqi Zhang, Tingyao Wu, Yingkang Yao, Xuedong Luo

**Affiliations:** 1 Faculty of Engineering, China University of Geosciences, Wuhan 430074, China; jiangnan@cug.edu.cn (N.J.); yuqiz@cug.edu.cn (Y.Z.); wutingyao@cug.edu.cn (T.W.); lxd328@163.com (X.L.); 2 Hubei Key Laboratory of Blasting Engineering, Jianghan University, Wuhan 430056, China

**Keywords:** blasting vibration, saturated soft soil, HDPE water supply pipeline, field test, numerical simulation, control vibration velocity

## Abstract

A key aspect of urban blasting engineering is evaluating the safety of the blasting dynamic load on the adjacent high-density polyethylene water supply pipeline and controlling the negative impact of the blasting vibration load on the pipeline. According to the special characteristics of the soil layer in Shenzhen coastal city, a field blasting test of a full-scale pre-buried HDPE pipeline was carried out, and the distribution characteristics of the blasting vibration velocity and dynamic strain were analyzed. The finite element model was established by LSDYNA, and the reliability of the calculation model and parameters was verified by comparing with the field test data. At the same time, the dynamic response characteristics of pipelines with different buried depths, operating water conveyance pressures, and diameters under blasting vibration loads were studied. Combined with the circumferential allowable stress control criterion of the pipeline, the safety control standard of the blasting vibration velocity of the HDPE water supply pipeline under different working conditions was proposed. The results show that the circumferential compressive strain of the HDPE pipe is the most affected by blasting vibration, and the pipe with the shortest blasting center distance has the largest response. The vibration velocity and equivalent stress of the pipeline increase with the increase of buried depth, internal pressure, and diameter. The vibration velocity and equivalent stress of the explosion side at the same section of the pipeline are greater than those of the back explosion side. Based on the dimensionless analysis, the vibration velocity prediction model of the buried depth, operating pressure, and diameter of the pipeline is proposed. The safety control velocity of the pipeline is 25 cm/s, and the vibration velocity of the upper surface is 22.5 cm/s according to the Mises yield strength criterion.

## 1. Introduction

High-density polyethylene (HDPE) pipeline has been widely used for urban water supply and drainage engineering due to its excellent performance. The overlying soft soil in coastal areas is mostly saturated. The effective stress is less than that of unsaturated soft soil under the same conditions, and the strength is lower and the deformation is easier. The buried pipeline buried in this stratum is vulnerable to excessive deformation caused by external load. Once the water supply pipeline is damaged, the life of urban residents will be seriously affected. Pipeline failure is mainly divided into two modes: one is static mode, such as ground permanent deformation caused by earthquakes. The other is the dynamic failure mode and the influence of seismic effect, including the blasting earthquakes caused by natural earthquakes and human activities. With the rapid development of the coastal urban economy, many urban underground space projects are constructed, often crossing hard rock. If the hard rock stratum is encountered in the process of rock and soil excavation, blasting fragmentation is often used for construction. How to control the influence of blasting vibration on the adjacent water supply pipeline in the blasting construction process and ensure the safety of the pipeline is the key concern. The study of the influence of the blasting load on the HDPE water supply pipeline buried in saturated silty clay stratum and the control standard of vibration failure of buried HDPE pipeline have theoretical research value and engineering practical significance.

At present, many scholars have carried out relevant research on the influence of vibration load on adjacent pipelines [1,2]. Ha [3] used a centrifuge test to study the deformation law of HDPE pipeline and combined it with the stress and strain data to obtain the relationship between the lateral force and deformation of the pipeline. Abdoun [4] studied the mechanical properties of HDPE pipes with different buried depths and diameters by a centrifuge test. Wang [5] conducted indoor similar model tests to study the vibration characteristics of rock mass and adjacent buried pipelines during the construction of a subway tunnel by the drilling and blasting method. As for field tests of pipeline response characteristics under blasting vibration, Zhu [6] proposed the dynamic response of a ductile iron gas pipeline under blasting vibration by field test. Xia [7] studied the dynamic response characteristics of the socket concrete pipeline through field tests and put forward the vibration velocity safety criterion of the pipeline. Zhong [8] obtained the dynamic response of polyethylene pipeline under explosive load by using field model tests. In addition, many scholars have used numerical simulation methods to study the dynamic response of buried pipelines [9,10]. Francini [11] used blasting numerical calculation to study the vibration law of adjacent buried pipelines and the surface above them, and they proposed corresponding safety criteria. Jiang [12] studied the dynamic response of a gas pipeline undercrossing tunnel blasting by field monitoring and LS-DYNA. Xia [13] obtained through field monitoring and LS-DYNA numerical simulation that the vibration velocity and equivalent stress of concrete pipeline under a full water state and blasting vibration load were smaller than those under the empty pipe state. As for numerical calculation, Kai Wu [14] used numerical simulation software to simulate the failure behavior of PE80 pipeline affected by factors such as pipe diameter, pipe wall thickness, internal pressure, tooth size, and excavation location during the excavation of a hydraulic excavator. Zhang [15] studied the dynamic response characteristics of buried concrete pipeline near blasting by using monitoring data.

Unlike theoretical and numerical simulation, field tests provide the most effective data [16,17,18]. In many studies, the effects of impact loading on pipelines are mainly based on theoretical and numerical simulation, and it is rarely possible to carry out full-scale model tests. During a full-scale test, many dynamic response data can be measured, such as vibration velocity, strain, displacement, etc. [19,20]. Among these monitoring programs, strain measurement and vibration velocity measurement, which take into account the force state and kinematics of the pipeline, are among the most attractive and widespread techniques. At present, most of the research on the dynamic response of pipelines is focused on concrete, cast iron, and other pipes with a hard texture, while less research has been done on the softer, tougher HDPE pipes. Meanwhile, the effect of near-blast vibration loading on buried water pipelines in saturated soft soil layers in coastal cities has been less studied.

In this study, a method of pipeline safety evaluation is proposed using the dimensionless analysis method to predict the vibration control rate for pipeline safety under blast loading by using vibration and strain data. Firstly, based on a typical buried water supply pipeline system with saturated soft soil in the Shenzhen coastal metro project, the response characteristics of HDPE water supply pipeline under blasting vibration loads with different pipe diameters, burial depths, and working pressures were analyzed by field blasting tests and numerical simulations. Second, by combining the dimensionless analysis method and the circumferential allowable stress control standard of a pipe, the controlled vibration velocity of the safe state of the pipe is obtained. Finally, the safety performance of the pipeline was evaluated comprehensively by the Mises stress criterion. The results show that the proposed method is feasible and effective.

## 2. Generalization of Coastal Metro Project

### 2.1. Brief Introduction of Coastal Metro Engineering and Construction Method

**Figure 1:**
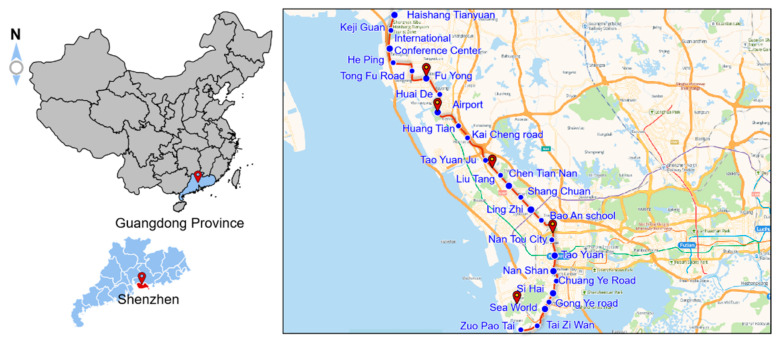
Location of Shenzhen city and map of Metro Line 12.

Shenzhen is a coastal city in southern China that is located in southern Guangdong, the eastern coast of the Pearl River Estuary, adjacent to Hong Kong; the geographical location is shown in Figure 1. The surface water along the line is mainly characterized by sea areas, rivers, ponds, ditches, and so on, and the surface water is more developed. The overlying strata are mainly composed of silt, silty clay, clay, and other soft soil, and the underlying bedrock is mainly composed of mixed granite and Sinian metamorphic rock.

Line 12 is the backbone line of Shenzhen rail transit from south to north in the recent construction plan of the Shenzhen rail transit network. It is a common speed line that supports the construction of the western development axis of Shenzhen and the urban development of the Qianhai (Shekou) Free Trade Zone and Airport New Town. Line 12 starts from the left artillery station and ends at the Haishangtianyuan east station, with a total length of about 41.18 km. The first-stage project planning of Line 12 is shown in Figure 1. The underground station of the line is constructed by the open-cut method, and the interval tunnel is constructed by the shield method or mining method. When the tunnel structure passes through the medium-to-micro weathered bedrock layer, or the floor of the tunnel structure is close to the medium-to-micro weathered bedrock surface, the mining method is construction. When the tunnel structure passes through the soil layer and soft rock stratum, the shield method is adopted. The open-cut method can be used for the sections with a relatively open site and shallow buried depth of interval tunnels. In the process of blasting and tunnel excavation using the mine method, short footage, bench excavation, and timely support should be adopted. It is not suitable for large-section excavation to avoid large collapse and safety accidents.

### 2.2. Characteristics of Buried Pipelines near Coastal Metro

**Figure 2:**
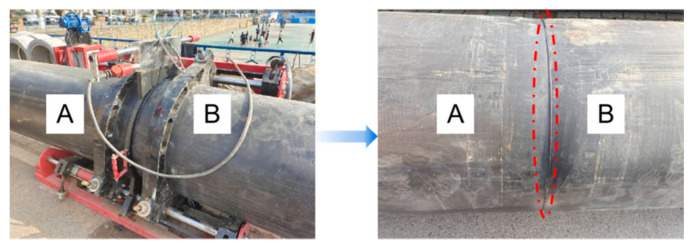
HDPE pipeline hot melt connection.

The project is mainly laid along the current road, and the ground environment is mainly traffic arteries, residential living areas, and commercial areas. The surface traffic is busy, and the buildings are dense. Along the road, the ground underground pipelines are dense, mainly including sewage pipelines, water supply pipelines, telecommunications pipelines, power pipelines, communication cables, and so on. The buried depth of underground pipelines is about 0.5 m to 3.0 m, and some old urban areas and streets are more than 5.0 m due to age. According to the engineering geological data, the overlying soil layer of the pipeline in the coastal subway project area is mostly saturated clay. The soil has high moisture content and low strength, and its dynamic shear modulus and damping ratio are greatly affected by the moisture content. The dynamic characteristics of seismic wave propagation in the soil are greatly different from those in the rock medium. HDPE pipe is a flexible pipe. When the stratum is deformed by external load, it is easy to cause damage to this type of pipe. Since the urban municipal pipeline systems are mostly long-distance transportation, different pipeline connection methods are often used according to different pipeline types and materials to facilitate transportation and installation. The commonly used pipeline connection methods are socket, sleeve, electrofusion, hot fusion, etc. In the small-diameter HDPE water supply pipe system, hot melt has been widely used in pipe connection due to its excellent physical properties and relatively simple connection mode. The connection form is shown in Figure 2. According to the specification, the working pressure at the interface of the hot melt pipeline is greater than that at the pipe body, which is higher in strength and more difficult to be destroyed.

### 2.3. Full-Scale Blasting Test near the HDPE Pipeline

Due to the extensive application of HDPE pipeline in urban water supply pipeline engineering, based on the blasting engineering of the Shenzhen Metro Line 12 subway tunnel under construction, the blasting test was carried out in a test site in Shenzhen, where the overlying soil layer was saturated with miscellaneous fill, cohesive soil, and the bedrock was granite. The main strata and parameters are shown in Table 1. The comparative analysis shows that the parameters of the silty clay layer in the site are similar to those of saturated silty clay layer in Shenzhen, which has certain representativeness. The lower blasting rock layer is close to the rock mass excavated in the actual blasting engineering.

According to the overburden characteristics of the buried pipelines near Shenzhen Metro Line 12, the test site was selected as the silty clay layer, and the buried depth of the pipeline (from pipe top to ground) was 2.0 m. The outer diameter (*D*) of the HDPE pipe was 60 cm, the inner diameter (*d*) was 49 cm, the wall thickness was 5.5 cm, and there was a smooth inner and outer wall. The pipeline material was PE100 grade, and the mechanical parameters of the pipeline are shown in Table 2. The trench size was 6 m long, 1 m wide, and 3 m deep. The hole diameter was 90 mm, using #2 rock emulsion explosive and non-conductive detonator blasting. The diameter of the coil was 70 mm, and the length was 350 mm.

Due to the limitation of the test site, only the blasting vibration test was carried out on the HDPE without pressure air pipe. Tests for pressurized water filling pipelines will be conducted later.

**Figure 3:**
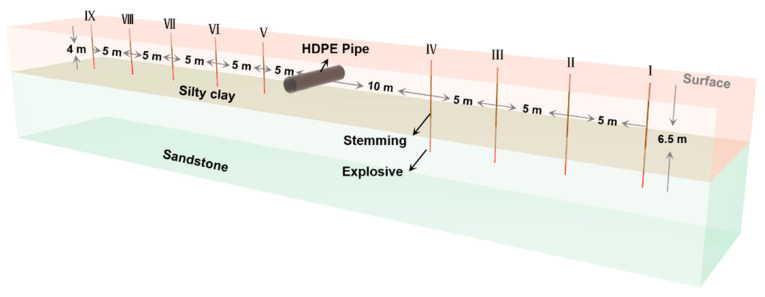
Layout diagram of the blasting hole.

**Figure 4:**
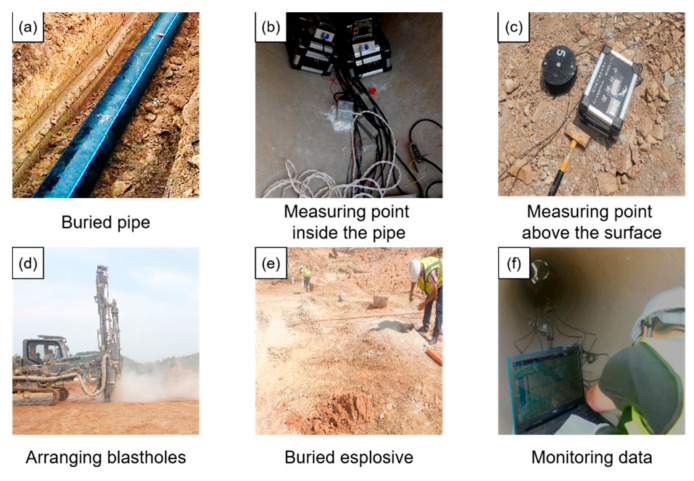
Field test process.

The working parameters of the specific explosive quantity, burial depth, and horizontal distance in each blasting test are shown in Table 3. The field test and schematic diagram of some blast holes are shown in Figure 3. The field test process is shown in Figure 4. The length of 8 kg explosive is 1 m, and the length of 9.6 kg is about 1.1 m.

### 2.4. Monitoring Sensors and Systems

#### 2.4.1. Vibration Test System

According to the experimental design scheme, in order to study the dynamic law of the blasting vibration effect of HDPE pipe, the dynamic test instrument was used to measure the soil layer in and around the pipe section, and the relevant data were dynamically measured in real time during the blasting experiment. The main test items in the experiment include the study of pipeline dynamic strain, pipeline particle vibration velocity, and surface vibration velocity above the pipeline. In order to study the vibration velocity of the buried pipeline and the surface above the pipeline, the blasting vibration test instruments in this test named TC-4850 were selected to monitor the vibration of particles for the pipeline and ground surface in different directions. According to the demand, multiple monitoring sections were set inside the pipeline and the surface above the pipeline to arrange multiple vibration velocity measuring points, and D1 to D4 is the vibration measuring point. The number of test channels of the TC-4850 instrument is three parallel channels, and the sampling rate is from 1 kHz to 50 KHz; also, it can trigger recording continuously and can record 128–1000 times. Moreover, its recording accuracy can be as low as 0.01 cm/s. Meanwhile, the data of PPV in the work of Lu S (2015) [21] and Nan (2018) [22] are also obtained by this type of monitoring instrument, and the research using the field tests of Lu (2015) [23] and Xia (2018) [24] are also based on the data obtained from this type of vibration monitoring instrument. What is more, the vibration monitoring instrument used in this paper has been regularly calibrated by a professional appraisal institution in China. The sensor converts the velocity of the seismic wave caused by blasting into a voltage signal, which is converted into a digital signal through a converter and recorded into the memory in the instrument. After the test is completed, the data line is transferred to the computer, which calculates and processes the signal, and it finally outputs it in the form of a report to the printer or stores it on the hard disk. The process of testing vibration data with a blast vibration tester is as follows.

(1) Signal input

The signal input interface uses a three-way signal input interface. After the sensor converts the physical quantities in the field into electrical signals, they are input into the instrument through the signal input interface.

(2) Signal Conditioning Circuit

The signal conditioning circuit is the bridge between the sensor and the converter, and it is an important part of the system. Its main function is to control the amplification of the signal conditioning circuit through the program.

(3) Analog electrical signal—digital signal converter

This part is the core part of the system and uses a 16-bit resolution converter. It converts the conditioned analog electrical signal into a digital signal so that the instrument can store the signal in the memory.

(4) CPU

The CPU completes the tasks of data reading, processing, and logic control of the system, data transmission, etc. In this system, we use a microcontroller with convenient control.

(5) Data Memory

The TC 4850 data memory provides the instrument with a place to store signals, with 128M storage space, using a maximum of 1024 KB data segments. A maximum of 1000 segments of data can be stored, and the data length can be set flexibly according to the actual situation. A maximum of 163 s of data files can be recorded (depending on the sampling rate).

(6) Clock Circuit

This instrument comes with a clock circuit. The CPU will automatically record the current clock at the moment of the blasting event, instead of the traditional human to record, which saves manpower to a greater extent.

(7) Signal output interface

The signal output of this system uses a high-speed USB2.0 interface. USB is a common computer communication interface with the characteristics of convenient connection. When the sensor output signal reaches the trigger level we set, the trigger control circuit starts the acquisition, the signal after the signal conditioning circuit enters the converter, and the CPU stores the acquired waveform data into memory. At the same time, the current time is recorded. Then, the data are transferred to the instrument’s memory and transmitted to a computer for analysis and processing via a multifunctional data line.

(8) Trigger

The trigger function represents the ability to capture signals and to trigger and acquire data based on many different conditions.

#### 2.4.2. Strain Testing System

In order to study the dynamic strain of the pipeline during blasting vibration, strain gauges in different directions were pasted on the inner surface of the pipeline. The strain gauge used in the experiment was uniaxial 80 mm, 120 Ω strain gauge, which was connected with a DH5956 dynamic signal acquisition instrument through the connection line. The DH5956 is a synchronous parallel high-speed data acquisition device with a 16-bit high-precision converter, a maximum sampling rate of 1 MSps for channel connection, and an on-board cache of up to 2G, which can realize real-time recording of multi-channel high-speed dynamic signals. The device can be connected to sensors with different interfaces for the corresponding signal measurement. Resistance strain gauges are used for strain measurement. The resistive strain gauge is made of 0.02–0.05 mm of copper or chrome wire wound into a grid sandwiched between two layers of insulation in the wafer (substrate). The measurement principle of resistance strain gauge is also considered: the resistance of a wire is not only related to the properties of the material but also to the length and cross-sectional area of the wire. The wire is affixed to the member, and when the member is deformed by force, the length and cross-sectional area of the wire change accordingly to the amount of mechanical deformation (tension or compression) to which it is subjected, resulting in a change in resistance.

**Figure 5:**
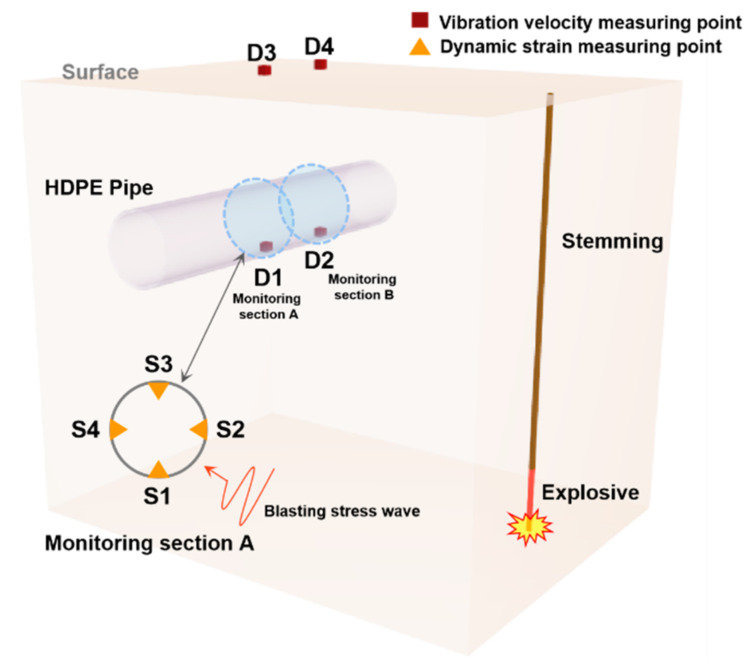
Test points of vibration velocity and dynamic strain.

**Figure 6:**
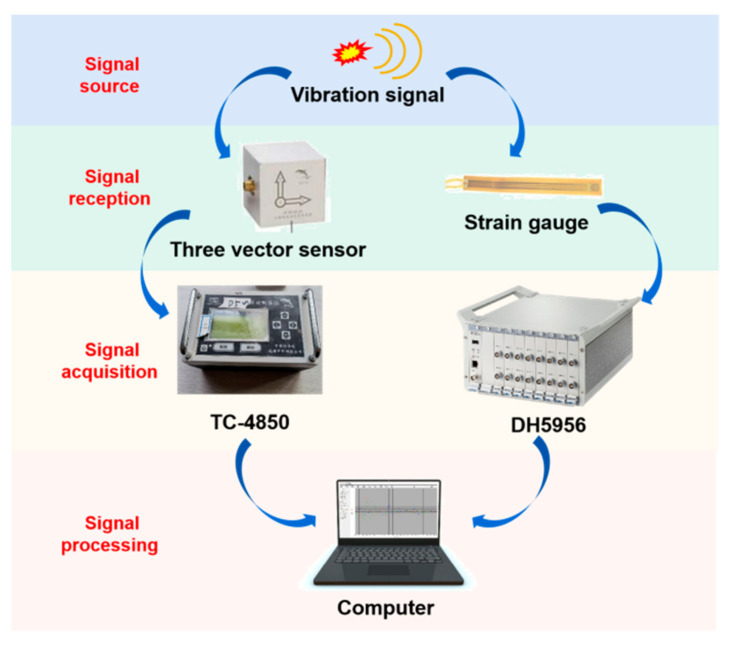
Monitoring system.

The use temperature of the monitoring instrument is generally stored at −40 to 60 °C, while in the limit use condition is −10 to 50 °C; its normal working range is 0 to 40 °C. The temperature for the use of the monitoring instrument is 40 °C at the normal operating range. The range of frequencies subjected to vibration during the use of the monitoring instrument is 5 to 55 Hz, at a drive amplitude (peak) of 0.19 mm. Its sweep rate is less than or equal to 1 octave/min; the holding time at the resonance point is 10 min, and the monitored vibration direction is in the x-direction and y-direction as well as the z-direction. The real-time dynamic data are collected during the blasting experiment, and the sampling frequency was set to 20 kHz. The arrangement of measuring points for pipeline vibration velocity and dynamic strain is shown in Figure 5. The monitoring system is shown in Figure 6. The monitoring section A was located on the symmetric surface of the pipeline center; that is, the distance between the nozzle section and section A was 3 m.

### 2.5. Analysis of Test Results

#### 2.5.1. Analysis of Vibration Velocity Test Results

The vibration velocity is the most intuitive measurement data in vibration monitoring: the energy of the blasting seismic wave passing through the medium. According to the above test scheme, the blasting vibration test data under nine working conditions were obtained. The statistics of the peak combined vibration velocity of each measuring point under nine working conditions and the maximum vibration velocity corresponding to the main frequency in three vector directions are shown in Table 4. According to the statistical results in Table 4, the peak vibration velocity increases with the increase of dosage and decreases with the increase of distance, which conforms to the attenuation law of blasting vibration wave.

According to the statistical analysis, the minimum main frequency measured in this experiment is 18 Hz, and the maximum main frequency is 124 Hz. The main frequency of blasting vibration is mainly concentrated between 10 and 50 Hz. Compared with the natural frequency of the buried pipeline (generally lower than 10 Hz), the vibration frequency of the test blasting seismic wave is less at low frequency, and the blasting energy is mainly concentrated in the high-frequency band. Therefore, the blasting seismic wave makes the pipeline structure have less resonance probability, which is beneficial to the blasting safety of the pipeline.

#### 2.5.2. Analysis of Dynamic Strain Test Results

**Figure 7:**
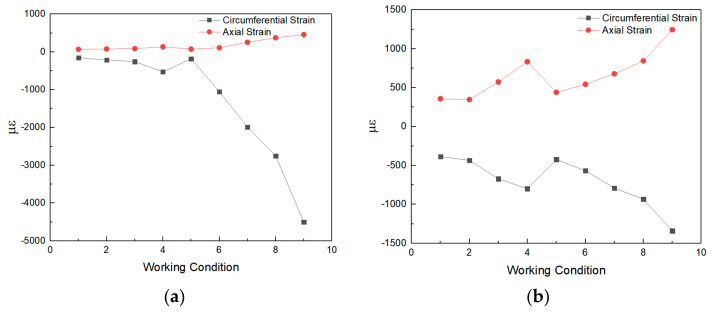

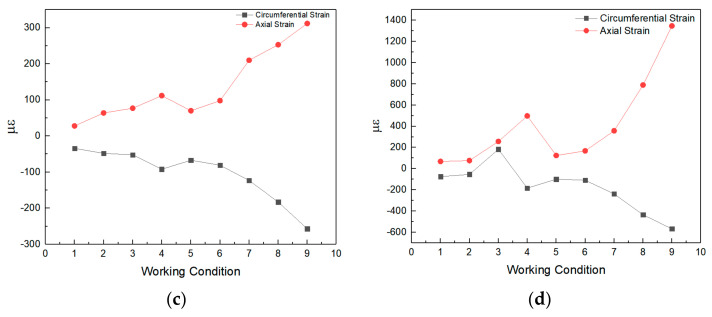
The peak strain of each measuring point in the monitoring section: (**a**) Measuring point 1; (**b**) Measuring point 2; (**c**) Measuring point 3; (**d**) Measuring point 4.

Since the measurement of strain data in the process of blasting experiment will be interfered by electromagnetic, noise, and other external factors, according to the dynamic experimental data measured by the DH5956 strain test system, the dynamic strain data measured by the experiment were denoised and filtered by the MATLAB program, and the peak strain data of blasting experiment under nine working conditions were analyzed. According to the above data processing method, the statistics of axial and circumferential dynamic strain peak values of each measuring point on the pipeline monitoring section under different working conditions are shown in Figure 7.

According to the results in Figure 7, the peak values of dynamic strain at each measuring point on the dangerous section of the pipeline show a law of increasing with the decrease of blasting distance and the increase of explosive quantity. It can be seen from Figure 7 that the axial peak strain of the pipeline on the explosion side is dominated by tensile strain, the axial peak strain on the back explosion side is dominated by tensile strain, and the maximum axial tensile strain appears at point 4. The circumferential peak strain of the pipeline at the explosion side is mainly compressive strain, and the maximum circumferential compressive strain appears at the explosion side of point 1. Combined with this experiment, the relevant physical and mechanical properties of HDPE were studied, and the tensile strength was greater than the compressive strength. According to the failure characteristics of HDPE pipeline, it can be seen that under blasting seismic load, the pipeline in this experiment is more vulnerable to damage due to excessive circumferential compressive stress [25,26].

## 3. Numerical Modeling of Dynamic Calculation and Verification for Buried HDPE Pipeline

### 3.1. Numerical Modeling of Dynamic Calculation for Buried Pipeline

**Figure 8:**
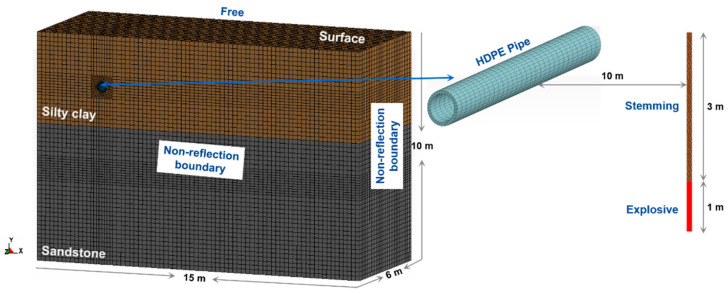
Overall model and mesh subdivision.

In practical engineering, due to the different laying time and use demand, the buried depth, inner and outer wall diameter, and operating pressure of the pipeline are mostly different. In order to study the dynamic response of water supply HDPE pipelines under blasting vibration load under different working conditions, LS-DYNA software was used to establish a numerical model. The explosive model and Jones–Wilkins–Lee (JWL) state equation of LS-DYNA software were used to simulate the dynamic response characteristics of a pipeline under empty pipe state under blasting vibration load. The overall size of the model was 15.0 m × 6.0 m × 10.0 m, the ratio of the pipe size to the site was 1:1, and the diameter of the blast hole was 90 mm. In the model, the buried pipeline soil was homogeneous saturated clay, and the bedrock was weathered granite. Due to the limited size of the model, except for the upper surface of the model, which was a free surface, other surfaces were non-reflective boundaries. The contact form between the pipeline and silty clay was surface contact, and the interface between the pipeline and liquid was a fluid–solid coupling interface. There was a common node contact form between the explosive grid and surrounding rock and soil mass, and there was adaptive grid division of the surrounding rock and soil mass. When establishing the water supply pipeline model under water-filled operation, the water inside the pipeline can be filled and pressurized on the inner wall of the pipeline. The established calculation model and its grid are shown in Figure 8. According to Regulations [27], a PE100 grade polyethylene pipe was selected, and SDR11 was selected according to the specification. Therefore, combined with the actual project and regulations, different operating parameters are shown in Table 5.

(1) Material model of saturated clay

The material model of silty clay is DRUCKER_PRAGER. The yield surface of the Mohr–Coulomb criterion in the partial plane is a hexagonal cone, and MAT_DRUCKER_PRAGER can construct a smooth yield surface without a corner tangent to the hexagonal cone of M-C criterion, which is beneficial to numerical calculation. The mechanical expression of the material model is:

(1) $$f=\alpha I_{1}(\sigma_{ij})+\sqrt{I_{2}(S_{ij})}+k=0.$$

where *f* is the plastic potential function; *I*_1_(*σ_ij_*) is the first invariant of stress tensor; *I*_2_(*σ_ij_*) is the second invariant of stress deviator tensor; *α* and *k* are the material constants, which are functions of material parameters *c* and *φ*, and *c* and *φ* are the cohesion and internal friction angles of materials, respectively. The specific input parameters are shown in Table 6. In Table 6, *ρ* is density, *E* is elastic modulus, *G* is shear modulus, *μ* is Poisson’s ratio, *c* is cohesion, *φ* is friction angle, and *σ*_t_ is tensile strength.

Weathered granite

To measure strong weathered siltstone and stemming, we used the PLASTIC_KINEMATIC material model [28,29,30,31]. The stress–strain relationship of the material model keywords is shown in Equation (2).

(2) $$\sigma_{Y}=\left[1+\left({\frac{\varepsilon }{C}}^{\frac{1}{P}}\right)\right]\left(\sigma_{0}+\beta E_{P}\varepsilon_{P}^{\mathrm{eff}}\right).$$

where *σ*_*Y*_ is the yield stress; *σ*_0_ is the initial yield stress; *ε* is the strain rate; *C* and *P* are variable parameters; $\varepsilon_{P}^{\mathrm{eff}}$ is the effective plastic strain; *E*_*P*_ is the plastic-hardening modulus; *β* is the hardening coefficient. The specific input parameters for calculation are shown in Table 7.

HDPE pipeline

HDPE material is a kind of viscoelastic material, and its mechanical properties are affected by four factors: stress, deformation, temperature, and time. The influence of temperature on HDPE material is not considered here; that is, its mechanical properties are related to strain rate. Therefore, the material model can adopt PLASTICITY_POLYMER, which can simulate the dynamic response of polymer under a high strain rate. The specific input parameters during calculation are shown in Table 8.

Water

The water in the model is defined by the keyword *MAT_NULL, and the state equation is defined by the keyword *EOS_GRUNEISEN in the form of:

(3) $$P=\frac{\rho_{0}C^{2}\mu \left[1+\left(1-\frac{\gamma_{0}}{2}\right)\mu -\frac{\alpha }{2}\mu^{2}\right]}{{\left[1-\left(S_{1}-1\right)\mu -S_{2}\frac{\mu^{2}}{\mu +1}-S_{3}\frac{\mu^{3}}{{\left(\mu +1\right)}^{2}}\right]}^{2}}+\left(\gamma_{0}+\alpha \mu \right)E\;\;\;\;\;\mu >0.$$

(4) $$P=\rho_{0}C^{2}\mu +\left(\gamma_{0}+\alpha \mu \right)E\;\;\mu >0.$$

where *C* is the sound velocity in water, *μ* = *ρ*/*ρ*_0_ − 1; *ρ* is the density of water after disturbance; and *ρ*_0_ is the initial density of water. *E* is the specific internal energy; *γ*_0_ is the Gruneisen coefficient; *α* is the volume correction coefficient; *S*_1_, *S*_2_, and *S*_3_ are *V*_*S*_–*V*_*P*_ slope coefficients. The specific input parameters during calculation are shown in Table 7.

Explosive

The explosive material in the model is consistent with the No. 2 rock explosive used in the experimental site. The high-energy explosive material HIGH_EXPLOSIVE_BURN brought by LS-DYNA software is used to simulate the explosive model. The specific parameters of the explosive are shown in Table 8. The JWL equation can describe the relationship between the detonation pressure of explosives and relative volume and internal energy as shown in Equation (5).

(5) $$p=A(1-\frac{\omega }{R_{1}V})e^{-R_{1}V}+B(1-\frac{\omega }{R_{2}V})e^{-R_{2}V}+\frac{\omega E_{0}}{V}.$$

where *p* is the explosion product pressure, *V* is the explosion product relative volume, *R*_1_, *R*_2_, *ω*, *A*, and *B* are the explosive material parameters, *E*_0_ is the initial specific internal energy. The related parameters of explosive detonation products are shown in Table 3. In Table 8, *ρ* is density.

### 3.2. Numerical Model and Parameter Validation Analysis

**Figure 9:**
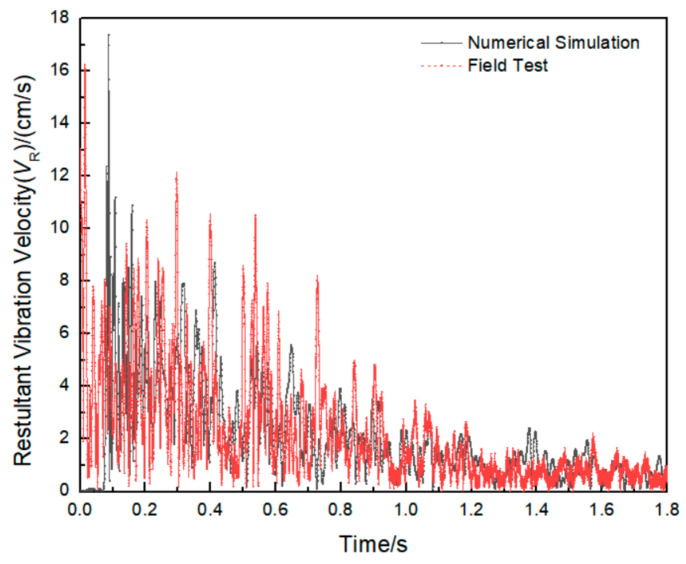
Comparison of resultant vibration velocity waveform between field test and numerical simulation.

In order to verify the accuracy of the established model and parameters, we used the same model as the actual working condition 8. The overall size of the model was 15.0 m × 6.0 m × 10.0 m, and the ratio of pipeline size to site was 1:1. There was no water and pressure inside the pipeline, which is the same as the actual field test. According to the field test monitoring scheme, the monitoring points were selected at the same position of the numerical model. The comparison between the numerical simulation results and the peak vibration velocity of the field measured data is shown in Table 9. In Table 9, *V*_RF_ and *V*_RN_ are the peak combined vibration velocity in three vector directions for the field test and numerical simulation, respectively, and *E*_VR_ is the combined vibration velocity error. The results of the combined vibration velocity on the pipeline monitoring section A are compared, and the comparison results are shown in Figure 9.

It can be seen from the results in Table 9 that there is little difference between the numerical simulation results and the field test results, and the maximum error is only 9.8%, which proves that the numerical simulation results and parameters are accurate and reliable.

### 3.3. Dynamic Response Analysis of Water Pipeline under Different Operating Pressures

**Figure 10:**
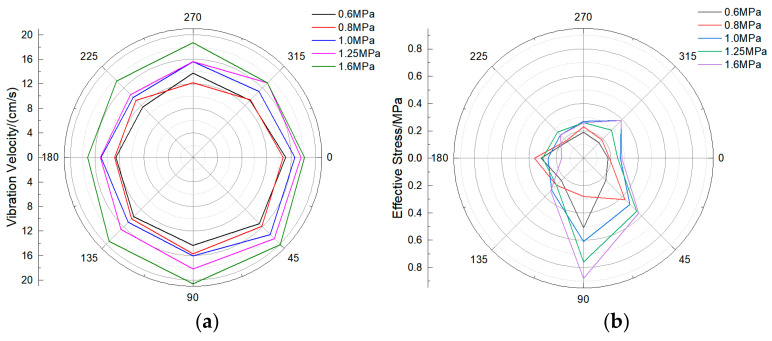
Vibration velocity and effective stress of pipeline section under different operating pressures: (**a**) Vibration velocity; (**b**) Effective stress.

Under different operating pressures, the pipeline response characteristics are also different. In order to study the influence of operating pressure on the response characteristics of pipelines, the size of the model pipeline is 60 cm in outer diameter, 55 mm in wall thickness, and 2 m in buried depth. The response characteristics under five different operating pressures are calculated, respectively. According to the calculation results, the response degree on the section with the closest distance from the pipeline to the explosive (i.e., the central symmetry plane of the pipeline) is the largest, so this section is used as the research section to analyze the results. The statistical results of the vibration velocity and effective stress of the same section are shown in the polar coordinate diagram. It can be found from Figure 10 that the vibration velocity and effective stress at 0–90° of the pipeline are greater than those at other positions. The maximum vibration velocity is located at 90° of the pipeline, and the maximum effective stress is located at 90° of the pipeline. In addition, with the increase of pipeline operating pressure, the vibration velocity of the pipeline is also significantly increased. When the operating pressure level is 1.6 MPa, the extreme values of maximum vibration velocity and effective stress appear in the burst test of the pipeline monitoring section A, reaching 20.61 cm/s and 0.88 MPa, respectively.

**Figure 11:**
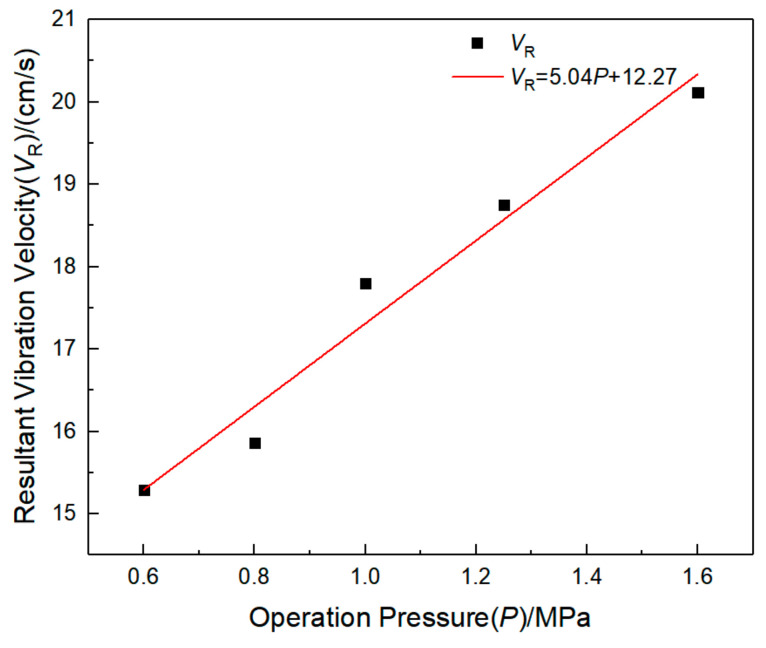
Fitting curve of operating pressure and peak resultant vibration velocity.

In order to study the characteristics of the surface vibration velocity above the pipeline, the maximum vibration velocity inside the pipeline, the surface vibration velocity above the pipeline, and the main frequency in each direction are counted, as shown in Table 10. It can be found that the surface vibration velocity above the pipeline also increases with the increase of pipeline operation pressure, and the vibration frequency in three directions also increases with the increase of pipeline operation pressure. This may be due to the existence of internal pressure, which increases the equivalent stiffness of the pipeline, while the increase of internal pressure increases the stiffness of the pipeline, and the response characteristics of the pipeline are more obvious. The main frequency of pipeline vibration is mostly concentrated about 50 Hz, which is much larger than the natural frequency of the pipeline. By fitting the maximum vibration velocity data of the pipeline with the operating pressure data, as shown in Figure 11, it can be found that there is a significant linear positive correlation between the vibration velocity and the operating pressure; *R*^2^ is 0.96.

### 3.4. Dynamic Response Analysis of Water Pipeline under Different Buried Depths

**Figure 12:**
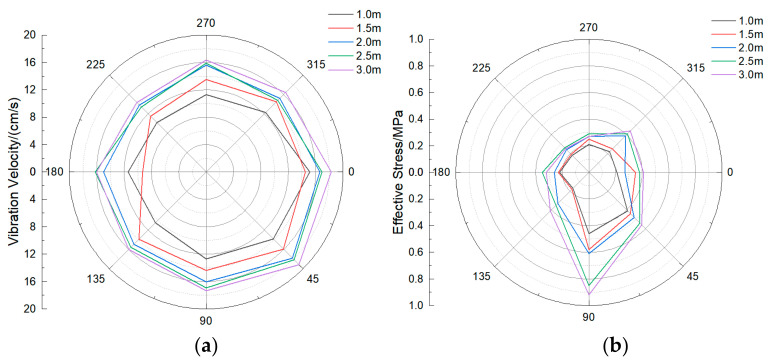
Vibration velocity and effective stress of the pipeline section under different buried depths: (**a**) Vibration velocity; (**b**) Effective stress.

Since the buried pipeline is crisscrossed and the buried depth is not the same, the buried depth also affects the pipeline response. Under different buried depths, the size of the model pipeline is 60 cm, the wall thickness is 55 mm, and the operating pressure is 1.0 MPa. The pipeline response characteristics under five buried depths are calculated, respectively. Similar to the previous section, the response degree on the section with the closest distance from the pipeline to the explosive (i.e., the central symmetry plane of the pipeline) is the largest, and this section is also used as the research section to analyze the results. The statistical results of the vibration velocity and effective stress of the same section are shown in the polar coordinate diagram. It can be found from Figure 12 that the vibration velocity and effective stress at 0–90° of the pipeline are greater than those at other positions. The maximum vibration velocity is located at 45° of the pipeline, and the maximum effective stress is located at 90° of the pipeline. In addition, when the location and diameter of the explosion source are constant, the vibration velocity and effective stress of the pipeline increase significantly with the increase of the buried depth of the pipeline. When the buried depth is 3 m, the maximum vibration velocity appears at the position of 45° in the pipeline blast test, and the maximum effective stress appears at the position of 90° in the pipeline blast test, reaching 17.8 cm/s and 0.63 MPa, respectively.

**Figure 13:**
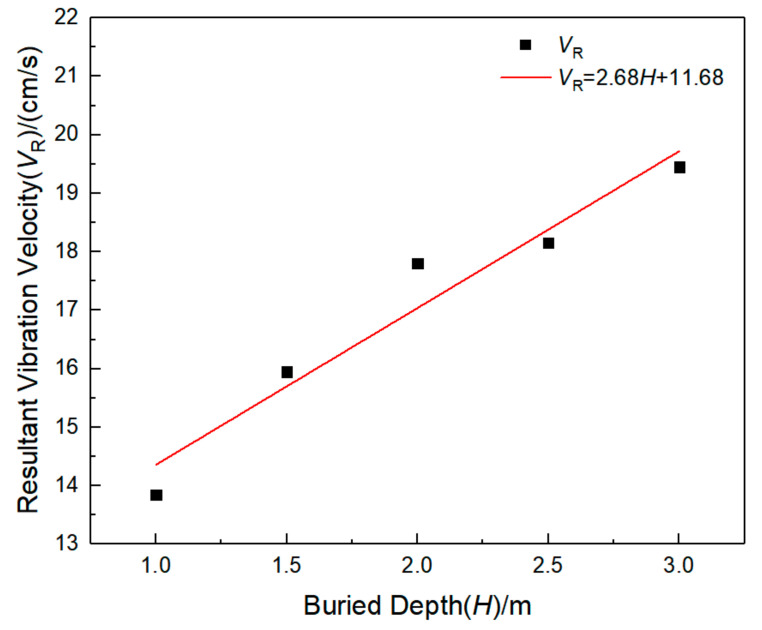
Fitting curve of operating pressure and peak resultant vibration velocity.

In order to study the characteristics of the surface vibration velocity above the pipeline, the maximum vibration velocity inside the pipeline, the surface vibration velocity above the pipeline, and the main frequency in each direction are counted, as shown in Table 11. It can be found that the surface vibration velocity above the pipeline also increases with the increase of the buried depth of the pipeline, and the vibration frequency in three directions also increases with the increase of the buried depth of the pipeline. The main frequency of pipeline vibration is mostly concentrated about 50 Hz, which is much larger than the natural frequency of the pipeline. This may be due to the explosion source position being unchanged; with the increase of buried depth, the pipeline from the explosion source position becomes shorter, so the degree of response increases, and the main frequency of vibration increases. Fitting the maximum combined vibration velocity data of the pipeline with the data of the buried depth of the pipeline, as shown in Figure 13, it can be found that the combined vibration velocity and buried depth have an obvious linear positive correlation, and the correlation coefficient *R*^2^ is 0.95.

### 3.5. Dynamic Response Analysis of Water Pipeline under Different Diameters

**Figure 14:**
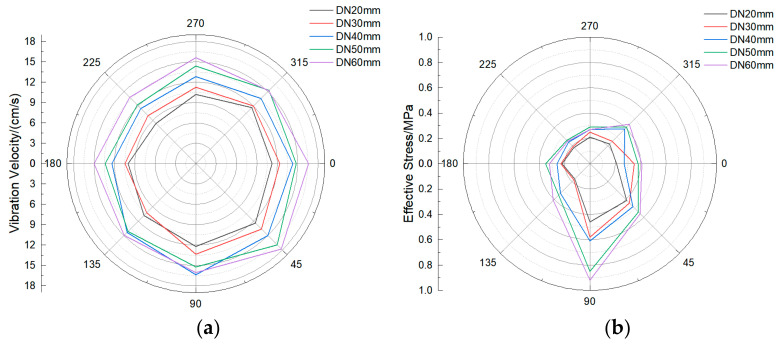
Vibration velocity and effective stress of pipeline section under different diameters: (**a**) Vibration velocity; (**b**) Effective stress.

In the urban water supply network system, different pipe diameters are applied to different water supply needs. Models were established under different pipe diameters, with a buried depth of 2 m and operating pressure of 1 MPa. The pipeline response characteristics under five pipe diameters were calculated. Similar to the previous section, the response degree on the section with the closest distance from the pipeline to the explosive (i.e., the central symmetry plane of the pipeline) is the largest, and this section is also used as the research section to analyze the results. The statistical results of the vibration velocity and effective stress of the same section are shown in the polar coordinate diagram. It can be found from Figure 14 that the vibration velocity and effective stress at 0–90° of the pipeline are greater than those at other positions. The maximum vibration velocity is located at 45° of the pipeline, and the maximum effective stress is located at 90° of the pipeline. In addition, when the location of the explosion source and the buried depth are constant, the vibration velocity and effective stress of the pipeline increase significantly with the increase of the pipe diameter. When the pipe diameter reaches 60 cm, the maximum vibration velocity appears at the 45° position of the pipe explosion test, and the maximum effective stress appears at the 90° position of the pipe explosion test, reaching 17.8 cm/s and 0.92 MPa, respectively.

**Figure 15:**
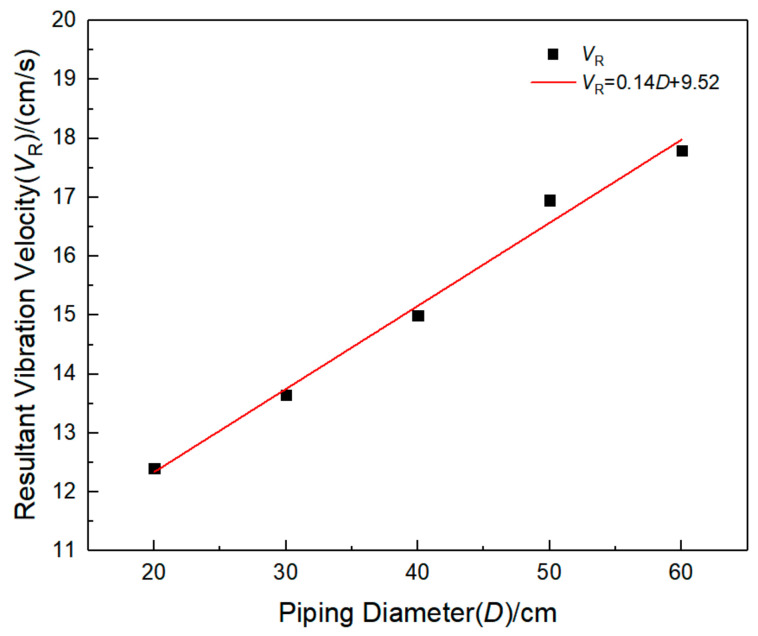
Fitting curve of operating pressure and peak resultant vibration velocity.

In order to study the characteristics of the surface vibration velocity above the pipeline, the maximum vibration velocity inside the pipeline, the surface vibration velocity above the pipeline, and the main frequency in each direction are counted, as shown in Table 12. It can be found that the surface vibration velocity above the pipeline also increases with the increase of pipe diameter, and the main frequency of vibration in three directions has little change with the increase of pipe diameter. The main frequency of pipeline vibration is about 50 Hz, which is much larger than the natural frequency of the pipeline. This may be due to the explosion source position being unchanged; with the increase of buried depth, the pipeline from the explosion source position becomes shorter, so the degree of response increases, and the main frequency of vibration increases. By fitting the maximum combined vibration velocity data of the pipeline with the diameter data, as shown in Figure 15, it can be found that the combined vibration velocity has a significant linear correlation with the buried depth, and the correlation coefficient *R*^2^ is 0.98.

## 4. Safety Criterion of Blasting Vibration Velocity of Buried Pipeline

In order to evaluate the safety performance of HDPE pipeline in water supply operation under blasting vibration load and calculate the safety control vibration velocity, the following assumptions need to be made: (1) The calculation assumes that the soil is linear elastic homogeneous, and there is no relative sliding between the pipe and soil under a blasting seismic wave. The pipe is nonlinear viscoelastic and satisfies isotropic. (2) The calculation results do not consider the influence of parameters such as void ratio and saturation of the soil layer. (3) The calculation object is the directly buried pipe body without considering the interface, bend, etc., because in accordance with the requirements of the specification, the weak links such as interface and bend need to be connected through flange, hot melt, electrofusion, etc., and its strength is greater than the pipe body part, so it is reasonable to calculate the control vibration velocity of the pipe body as the research subject.

Considering that the diameter, operating pressure, and buried depth of HDPE water supply pipeline are mostly different in practical engineering, many factors should be considered in calculating the safety criterion of buried HDPE water supply pipeline under blasting vibration load.

### 4.1. Dimensionless Analysis Models for Pipeline Safety Assessment

The attenuation of blasting seismic wave propagation in rock and soil is affected by the explosion source, the distance to the explosion source, and the working condition of the pipeline. The main variables involved in the propagation in rock mass media are summarized, as shown in Table 13. By dimensional analysis of Buckingham theorem (π theorem), the peak vibration velocity (*V*_P_) of pipeline particles can be expressed as:

(6) $$V_{P}=F(Q,r,H,D,P,\rho )$$

Combined with the above conclusions, the peak particle vibration velocity *v* of the pipeline is positively correlated with the buried depth *H*, diameter *D*, and operating pressure *P* of the pipeline, and it is positively correlated with the explosive quality *Q* and negatively correlated with the distance r from the measuring point to the explosion source. Therefore, according to the dimensional homogeneous theorem, the prediction formula of the peak particle vibration velocity of the pipeline can be preliminarily obtained as follows:

(7) $$V_{P}=k{\left(\frac{DH}{\rho^{2}r^{3}}\sqrt{\frac{QP}{r}}\right)}^{\alpha }$$

where *k* and *α* are the fitting parameters of the equation. For a certain field, *ρ* can be approximated as a constant. So, the formula can be expressed as:

(8) $$V_{P}=k{\left(\frac{DH}{r^{3}}\sqrt{\frac{QP}{r}}\right)}^{\alpha }$$

In order to fit the formula, the orthogonal experimental design was carried out with the parameters mentioned above, and the numerical model was established to supplement the previous working conditions. The orthogonal experiment table of three factors and three levels is designed. The buried depth of the pipeline is 1 m, 2 m, and 3 m. The diameter of the pipeline is DN20 cm, DN40 cm, and DN60 cm. The operating pressure of the pipeline is 0.6 MPa, 1.0 Mpa, and 1.6 MPa.

According to the above test rules, the vibration velocity measuring points are selected at the explosion test point of section A. Some of the working conditions coincide with the above model, and the results are counted as shown in Table 14.

Using software to fit the formula, *k* = 140.93, *α* = 0.29, *R*^2^ = 0.89, the fitting degree is high. The prediction formula of pipeline peak vibration velocity considering the influence of buried depth, pipe diameter, and internal pressure is as follows.

(9) $$V_{P}=140.93{\left(\frac{DH}{r^{3}}\sqrt{\frac{QP}{r}}\right)}^{0.29}$$

### 4.2. Safety Criterion of the Pipeline Based on the Prediction Model

**Figure 16:**
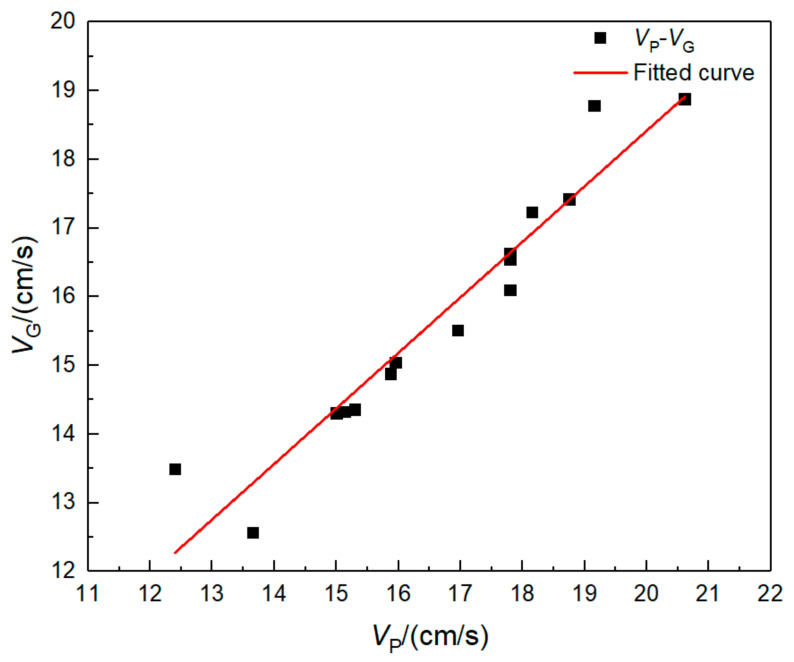
Fitting curve of peak vibration velocity between pipeline and ground.

Since the pipeline is located below the surface in practical engineering, it is difficult to directly monitor. In order to monitor the peak vibration velocity of the pipeline under blasting vibration, many scholars infer the vibration velocity of the pipeline by studying the vibration velocity of the surface above the pipeline, so as to monitor the safety of the pipeline. Accordingly, according to the above analysis of the peak combined vibration velocity (*V*_P_) of the pipeline and the peak combined vibration velocity (*V*_G_) of the surface above it, the data of the two are fitted, and the fitting results are shown in Figure 16. The fitting equation is as follows:

(10) $$V_{G}=0.808V_{P}+2.256$$

**Figure 17:**
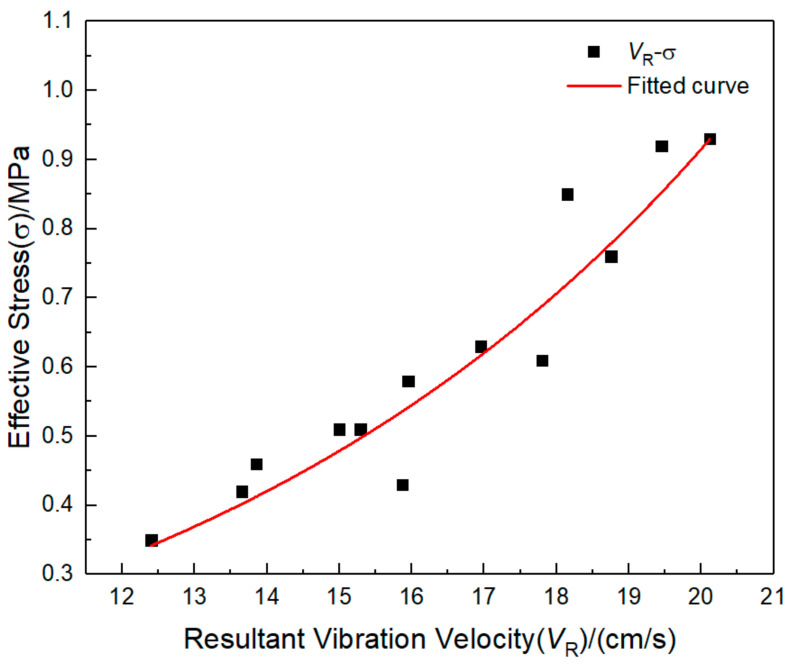
Fitting curve of peak vibration velocity and equivalent stress.

The peak combined vibration velocity and equivalent stress of the point with the maximum response to the pipeline blast test are fitted, as shown in Figure 17. In Figure 17, *σ* is the equivalent stress, and *V*_P_ is the peak combined vibration velocity of the pipeline. The equation of the fitting curve between the two is Equation (11), *R*^2^ = 0.93, and the fitting degree is good.

(11) $$\sigma =0.07\exp\left(0.13V_{P}\right)$$

where *σ* is pipeline peak equivalent stress, and *V*_*P*_ is pipeline peak vibration velocity.

In order to obtain the maximum control speed of HDPE corrugated pipe under blasting vibration during water-filling operation, the formula for calculating the maximum allowable working pressure of HDPE pipe given in the specification used. Under the normal working condition of the pipeline system, the maximum design internal water pressure (*F*_wd_) of the selected pipe should be 1.5 times the working pressure of the pipeline. In this project, *F*_wd_ is 2.4 MPa. The design value of the circumferential stress of the pipe wall under the action of internal water pressure (*σ*_θ_) can be calculated according to Equation (12). *SDR* = *D*_0_/*e*_n_ is defined in combination with the ratio of diameter to thickness. *SDR* = 11 is selected in this project, and the design value of circumferential stress of the pipeline wall is 15.84 MPa.

(12) $$[\sigma_{\theta }]=\frac{\gamma_{Q}F_{\mathrm{wd}}D_{0}}{2e_{n}}.$$

According to Tian [32], the stress generated under the action of internal pressure and external load for the thermal and power pipelines used in industry can be calculated according to the following formula through force analysis using the thin-walled cylinder principle, and the schematic diagram of the three stress directions is shown in Figure 8.

Hoop stress:

(13) $$\sigma_{\theta }=P_{js}r_{n}{}^{2}(1+r_{w}^{2}/r^{2})/(r_{w}^{2}-r_{n}^{2}).$$

Axial stress:

(14) $$\sigma_{L}=P_{sj}r_{n}^{2}(r_{w}^{2}-r_{n}^{2}).$$

Radial stress:

(15) $$\sigma_{r}=P_{js}r_{n}^{2}(1-r_{w}^{2}/r^{2})/(r_{w}^{2}-r_{n}^{2}).$$

Here, *σ*_*θ*_ is circumferential stress (MPa), *σ*_*L*_ is axial stress (MPa), *σ*_*r*_ is radial stress (MPa), *P*_*js*_ is calculation stress of internal pressure (MPa), *r*_*w*_ is the radius of the outer wall (cm), *r*_*n*_ is the radius of the inner wall (cm), and *r* is the radius of any calculation on the wall (cm). Therefore, it can be seen that if the hoop stress is a known quantity, then the hoop stress is used to represent the other two directions:

Axial stress:

(16) $$\sigma_{L}=\sigma_{\theta }/(1+r_{w}^{2}/r^{2}).$$

Radial stress:

(17) $$\sigma_{r}=\sigma_{\theta }(1-r_{w}^{2}/r^{2})/(1+r_{w}^{2}/r^{2}).$$

Considering that the failure mode of PE pipeline is mainly yield aging, the Mises yield criterion (Equation (18)) is used to evaluate [33]. When *r* = *r*_*w*_, the calculated yield stress is the smallest, so as to evaluate and judge the safety of the pipeline. Therefore, three principal stresses can be obtained according to the above formulas: *σ*_1_ = *σ*_*θ*_, *σ*_2_ = *σ*_*L*_, and *σ*_3_ = *σ*_*r*_ = 0. By substituting the three principal stress formulas in parallel with Equations (16) and (17) into Equation (18), the equivalent stress expressed in terms of cyclic stress can be obtained as *σ* = 0.866[*σ*_*θ*_].

(18) $$\sigma =\sqrt{\frac{1}{2}{\left(\sigma_{1}-\sigma_{2}\right)}^{2}+{\left(\sigma_{2}-\sigma_{3}\right)}^{2}+{\left(\sigma_{3}-\sigma_{1}\right)}^{2}}.$$

where *σ* is the effective stress (MPa), *σ*_1_ is the first principal stress (MPa), *σ*_2_ is the second principal stress (MPa), and *σ*_3_ is the third principal stress (MPa).

Equation (11) shows the equivalent stress and the peak vibration velocity at the dangerous section of the pipeline obtained by fitting; combined with the design value of the circumferential stress of the pipeline mentioned above [*σ*_*θ*_] = 15.84 MPa, it can be obtained that the safety control velocity of the pipeline in the operation state is 40.60 cm/s.

The pipeline materials used in engineering construction are mostly PE80 grade, and the mechanical parameters of the pipeline in this paper are taken from PE100 grade pipeline. When the mechanical test of the pipeline is carried out according to the specification, the pressure of the PE80 grade pipeline is less than that of the PE100 grade pipeline, and the strength reduction coefficient is 1.3. As a result of the problems of temperature and service life in practical engineering, the pipeline will be aging to a certain extent, and the minimum value of the aging coefficient is 0.8, according to the specification.

Therefore, the combined vibration velocity of safety control of the HDPE water supply pipeline in a water-filling state under the blasting vibration of this blasting project is 25 cm/s. Combined with the fitting formula of surface vibration velocity and pipeline vibration velocity, the surface safety control velocity can be selected as 22.5 cm/s.

## 5. Conclusions

Through the on-site pre-buried pipeline blasting test and monitoring, the numerical model is established to supplement the working conditions. Combined with theoretical analysis, the influence of blasting seismic load on the HDPE pipeline of buried operation water supply is studied. The main conclusions are as follows:

(1) Through field tests, it is found that when HDPE pipe is affected by blasting vibration, the circumferential compressive strain is the largest, and the pipe is more prone to compression failure in the circumferential direction. The main frequency of blasting vibration is mainly concentrated at about 50 Hz, but it is higher than the natural frequency of the pipeline.

(2) The combined vibration velocity and equivalent stress of the pipeline increase with the increase of pipeline diameter, buried depth, and operating pressure, and the combined vibration velocity and equivalent stress of the explosion side at the same cross-section of the pipeline are greater than those of the back explosion side.

(3) Through the data fitting analysis, the peak vibration velocity of the pipeline and the surface measuring point has a high linear correlation, and the peak vibration velocity of the pipeline has a curve correlation with the peak equivalent stress. Moreover, the peak combined vibration velocity in the pipeline can be calculated by the mathematical model prediction formula related to the pipeline diameter, buried depth, and operating pressure.

(4) Based on the numerical simulation results and relevant specifications, the safety assessment of buried operation water supply HDPE pipeline affected by earthquake damage is carried out. The calculated blasting vibration control speed should be 25 cm/s, and the surface control speed should be 22.5 cm/s, which provides an important reference for the seismic capacity analysis and safety protection of buried operation water supply HDPE pipeline affected by blasting.

## Figures and Tables

**Table 1:** Working condition parameter.

Stratum	Natural Weight-Specific Density (*ρ*)/(kN·m^3^)	Friction Angle (*φ*)/(°)	Cohesion (*c*)/(kPa)	Bearing Capacity (*fk*)/(kPa)
Landfill	19.2	18	8	120–160
Silty clay	19.3	12	25	160–180
Weathered granite	26.8	5.5	43	2000–4000

**Table 2:** Statistical table of adjacent blasting works.

Tubing	Elastic Modulus (*E*)/(MPa)	Density (*ρ*)/(g/cm^3^)	Ring Stiffness (*SN*)/(kN/m^2^)	Ultimate Strength (*σ*_u_)/(MPa)	Poisson Ratio (*μ*)
HDPE	834.9	0.936	8	31.6	0.46

**Table 3:** Working condition parameters of blasting test.

Working Condition	Burial Depth/m	Explosive Quantity/kg	Horizontal Distance/m
1	6.5	8	25
2	6.5	8	20
3	6.5	8	15
4	6.5	8	10
5	4	8	25
6	4	8	20
7	4	8	15
8	4	8	10
9	4	9.6	5

**Table 4:** Peak resultant vibration velocity and corresponding dominant frequency.

Working Condition	D1		D2		D3		D4	
	*V*_R_/(cm/s)	*f*/Hz	*V*_R_/(cm/s)	*f*/Hz	*V*_R_/(cm/s)	*f*/Hz	*V*_R_/(cm/s)	*f*/Hz
1	8.32	25	8.60	43	7.92	41	6.97	37
2	9.47	31	8.28	33	8.31	61	7.87	57
3	12.24	35	12.31	43	11.58	50	11.61	42
4	16.37	52	14.54	40	14.89	43	13.29	36
5	9.23	48	8.75	56	9.02	23	8.38	45
6	11.87	53	12.67	54	11.88	47	12.21	67
7	13.35	18	14.38	49	10.34	124	11.38	88
8	17.20	62	16.54	43	18.61	42	16.61	37
9	22.25	44	20.35	38	20.31	37	18.27	77

**Table 5:** Working condition parameters of numerical mode.

Working Condition Group	Internal Pressure (*A*)/MPa	Dimension (*B*)		Buried Depth (*C*)/m
		Diameter/mm	Wall Thickness/mm	
I	0.6	200	18	1
II	0.8	300	28	1.5
III	1.0	400	36	2
IV	1.25	500	45	2.5
V	1.6	600	55	3

**Table 6:** Pipeline, silty clay, and sandstone material model parameter table.

Material	*ρ*/(g/cm^3^)	*E*/GPa	*G*/GPa	*μ*	*c*/MPa	*φ*/(°)	*σ*_t_/MPa
Pipeline	0.936	0.8349	-	0.46	-	-	31.600
Soft layer	1.980	0.0390	0.027	0.35	0.035	15	0.028
Rock stratum	2.680	52.0000	11.26	0.25	5.500	43	2.580

**Table 7:** Working condition parameter.

Parameter	Density/(g/cm^3^)	Sound Velocity/(m/s)	*S*_1_	*S*_2_	*S*_3_
Value	1.0	1 500	2.56	1.986	1.2268

**Table 8:** Detonation product parameter table.

Parameter	*ρ*/(g/cm^3^)	*A*/GPa	*B*/GPa	*R*_1_	*R*_2_	*ω*	*E*_0_/GPa	*V*/cm^3^
Value	1.25	214	18.2	4.2	0.9	0.15	4.19	1

**Table 9:** Comparison results of field test and numerical simulation of combined vibration velocity.

Measuring Point	*V*_RF_ (cm/s)	*V*_RN_ (cm/s)	*E*_VR_
D1	16.37	17.41	6.35%
D2	14.54	15.98	9.90%
D3	14.89	15.66	5.17%
D4	13.29	14.57	9.63%

**Table 10:** Peak resultant vibration velocity of pipeline and surface.

Work Condition (A)		I		II		III		IV		V	
		Pipe	Ground	Pipe	Ground	Pipe	Ground	Pipe	Ground	Pipe	Ground
*V*_R_/(cm/s)		15.29	14.36	15.8	14.88	17.80	16.13	18.75	17.42	20.61	18.88
*f*/Hz	X	37	57	78	62	65	67	52	78	53	83
	Y	55	43	43	34	44	53	46	52	27	50
	Z	33	22	31	26	34	29	35	41	36	43

**Table 11:** Peak resultant vibration velocity of pipeline and surface.

Work Condition (B)		I		II		III		IV		V	
		Pipe	Ground	Pipe	Ground	Pipe	Ground	Pipe	Ground	Pipe	Ground
*V*_R_/(cm/s)		15.1S	14.33	15.95	15.04	17.80	16.55	18.15	17.23	19.15	18.78
*f*/Hz	X	57	45	48	76	54	79	50	83	62	67
	Y	43	51	56	64	31	66	33	72	47	71
	Z	24	32	43	36	45	52	25	60	65	55

**Table 12:** Peak resultant vibration velocity of pipeline and surface.

Work Condition (C)		I		II		III		IV		V	
		Pipe	Ground	Pipe	Ground	Pipe	Ground	Pipe	Ground	Pipe	Ground
*V*_R_/(cm/s)		12.4	13.5	13.65	12.57	15.0	14.31	16.95	15.51	17.80	16.63
*f*/Hz	X	49	55	53	81	61	81	55	73	41	72
	Y	56	45	46	54	41	54	55	62	35	43
	Z	31	43	29	31	39	42	43	46	44	64

**Table 13:** Important physical quantities involved in pipeline blasting vibration.

	Variable	Dimension
Dependent variable	Peak vibration velocity of pipeline particle/*v*	LT^−1^
Independent variable	Explosive quality/*Q*	M
	Distance from measuring point to explosion source/*r*	L
	Buried depth of pipeline/*H*	L
	Diameter of pipeline/*D*	L
	Pipeline operating pressure/*P*	ML^−1^T^−2^
	Density of rock and soil mass/*ρ*	ML^−3^

Note: L, T, and M represent dimensions of length, time, and quality, respectively.

**Table 14:** Statistical results of vibration velocity.

Working Condition	*D*/(cm)	*H*/(m)	*P*/(MPa)	*r*/(m)	*Q*/(kg)	*V*_P_/(cm/s)
1	20.0	1.0	0.6	11.14	8	10.25
2	20.0	2.0	1.0	10.73	-	12.25
3	40.0	3.0	1.0	10.38	-	19.30
4	20.0	3.0	1.6	10.41	-	17.65
5	40.0	1.0	1.6	11.09	-	12.85
6	20.0	2.0	1.6	10.73	-	15.60
7	60.0	2.0	0.6	10.66	-	15.29
8	60.0	2.0	0.8	10.66	-	15.86
9	60.0	2.0	1.0	10.66	-	17.80
10	60.0	2.0	1.25	10.66	-	18.75
11	60.0	2.0	1.6	10.66	-	20.12
12	20.0	2.0	1.0	10.73	-	12.40
13	30.0	2.0	1.0	10.72	-	13.65
14	40.0	2.0	1.0	10.70	-	15.00
15	50.0	2.0	1.0	10.68	-	16.95
16	60.0	2.0	1.0	10.66	-	17.80
17	60.0	1.0	1.0	11.05	-	13.85
18	60.0	1.5	1.0	10.85	-	14.95
19	60.0	2.0	1.0	10.66	-	17.80
20	60.0	2.5	1.0	10.50	-	18.15
21	60.0	3.0	1	10.36	-	19.15

Note: *D* represents diameter; *H* represents buried depth; *P* represents operating pressure; *r* represents blasting center distance; *Q* represents explosive quality; and *V*_P_ represents particle peak vibration velocity.

## Data Availability

The data presented in this study are available on request from the corresponding author. The data are not publicly available due to the ethical declaration issue.
